# Support Needs and Experiences of People Bereaved by Suicide: Qualitative Findings from a Cross-Sectional British Study of Bereaved Young Adults

**DOI:** 10.3390/ijerph15040666

**Published:** 2018-04-03

**Authors:** Alexandra Pitman, Tanisha De Souza, Adelia Khrisna Putri, Fiona Stevenson, Michael King, David Osborn, Nicola Morant

**Affiliations:** 1 UCL Division of Psychiatry, University College London, Maple House, 149 Tottenham Court Road, London W1T 7NF, UK; michael.king@ucl.ac.uk (M.K.); d.osborn@ucl.ac.uk (D.O.); n.morant@ucl.ac.uk (N.M.); 2 Camden and Islington NHS Foundation Trust, St Pancras Hospital, London NW1 0PE, UK; 3 North East London NHS Foundation Trust, Memory Service, Broad Street Health Centre, Morland Road, Dagenham, Essex RM10 9HU, UK; Tanisha.DeSouza@nelft.nhs.uk; 4 UGM Faculty of Psychology, Jl. Sosio Humaniora 1, Sleman, Universitas Gadjah Mada, Yogyakarta 55281, Indonesia; adelia.k.p@ugm.ac.id; 5 UCL Research Department of Primary Care & Population Health, Rowland Hill St, London NW3 2PF, UK; f.stevenson@ucl.ac.uk

**Keywords:** suicide, bereavement, support, grief, unmet needs, qualitative research

## Abstract

People bereaved by suicide are at increased risk of suicide, but evidence is lacking that available interventions reduce suicide risk. Few large-scale studies have described the views of suicide-bereaved people regarding their needs for support. Our objective was to explore the nature of young adults’ experiences of support after bereavement by suicide and their views on valued and unhelpful aspects. We conducted a cross-sectional study of staff and students aged 18–40 at 37 United Kingdom (UK) higher educational institutions in 2010, eliciting qualitative responses to two questions probing experiences of support and unmet needs after the suicide of a close contact. We conducted thematic analysis of responses from 420 adults bereaved by suicide, of whom 75% had received support after the loss. We identified three broad descriptive areas corresponding to important aspects of support: value and experiences of the support received; views on specific support needs; and reasons for not seeking support. We found that needs for emotional support exist throughout the social networks of people who die by suicide but are often hidden. Our findings suggest a need for proactive offers of support from family, friends, and professionals after suicide, repeated regularly in case a bereaved person does not feel ready for support early on.

## 1. Introduction

It is estimated that over 800,000 people throughout the world die by suicide each year [1] and that for approximately 45% of suicides, at least one suicide attempt has preceded it [2]. Previous attempt is regarded as the strongest risk factor for suicide [1], and international suicide prevention strategies place appropriate emphasis on interventions for people who self-harm. Many strategies also identify people bereaved by suicide as a vulnerable group, recommending increased support and information for family and friends after a suicide. However, the nature of indicated support in such recommendations remains unclear due to the lack of evidence for acceptability and effectiveness in reducing risk of suicide-related outcomes [3]. In England, a lack of progress in implementing support for families bereaved by suicide has been acknowledged [4], and there is also a need to address this internationally to reduce the risk of further suicides [5]. 

The inclusion of people bereaved by suicide in suicide prevention strategies is based on strong evidence from population registries that suicide risk is elevated in suicide-bereaved spouses [6,7], particularly men [6], and mothers [8]. Suicide bereavement is also associated with other adverse outcomes [9], including an increased risk of suicide attempt [10], drop-out from a college course or job [10], poor social functioning [10], and high levels of stigma, shame, guilt, and feelings of responsibility [11]. Survey findings suggest that 60 people are deeply affected by each suicide death [12] but estimates are as high as 135 [13]. The lifetime prevalence of losing a close friend or relative to suicide is estimated at 22%, with 4% past-year prevalence [14]. In one United States (US) survey, 51% of people in a general population sample were acquainted with one or more people who had died by suicide, and 35% of these people experienced moderate to extreme emotional distress following these deaths, even up to 14 years after the loss [15]. Whilst not all those who experience a close contact’s suicide will perceive negative health effects, evidence points towards a continuum of effects, in which perceptions of closeness to the deceased predict the likelihood of developing mental health problems after the loss [16,17].

It has been suggested that poor mental health outcomes after suicide bereavement may be related to the lack of support reported after suicide loss [18], with stigma implicated as one of the barriers to seeking or being offered help [19]. Quantitative survey work shows that one in four people bereaved by suicide receive no support after the death [20]. They are also significantly less likely to receive informal support after the loss than those bereaved by other causes of sudden death and significantly more likely to experience delays in receiving any support [20]. However, few studies have elicited the accounts of people bereaved by suicide in relation to their support needs and experiences [5,21]. Qualitative accounts from bereaved people suggest that responses by agencies are often insensitive and not aligned with the needs of families affected by suicide [22]. For their own part, general practitioners (GP) report a lack of training and confidence in how to respond after suicide, despite bereaved people viewing their GP as a key source of support [23,24]. 

There is a need to better understand what is perceived to be helpful and unhelpful after suicide bereavement, both to develop appropriate interventions and to improve the responses of professionals and the public in the aftermath of suicide. Our objective was to elicit the views of a national sample of young adults bereaved by the suicide of a close friend or relative, to explore the nature of their experiences of bereavement support and their suggestions regarding appropriate support provision. 

## 2. Materials and Methods 

### 2.1. Procedures

To collect data on the bereavement experiences of young adults, the age group of greatest policy interest at the time of designing the study [25], we invited all adults aged 18–40 who were working or studying at United Kingdom (UK) higher education institutions (HEIs) to participate in a closed, online study about sudden bereavement: the UCL Bereavement Study. This upper age limit was higher than the age limit of 24 used by the WHO for young adults [26] to avoid collecting only recent experiences of young bereaved adults. 

As described previously [10], we sampled via the email systems of large institutions as a means of accessing hard-to-reach groups, particularly men. This avoided the biases associated with recruiting a help-seeking sample [27], such as a bereavement support group. Our approach offered unique access to a large, varied but defined sample of young adults, including those at universities and agricultural, art, and drama colleges (all listed under acknowledgements).

Recruitment took place in 2010. We invited all 164 HEIs in the UK in 2010 to participate. Over 20% (37/164) agreed to take part, with a higher response (40%) from the group of universities characterised by higher research income. All 659,572 staff and students in this sampling frame received an individual email inviting them to take part in a survey of “the impact of sudden bereavement on young adults”, including an embedded survey link. Ten HEIs varied this approach by emailing students only, including the invitation within their weekly newsletter email, or advertising it on the intranet. 

### 2.2. Inclusion Criteria

Inclusion criteria were: adults aged 18–40 who, since the age of ten, had experienced sudden bereavement of a close friend or relative. We excluded early childhood bereavements to minimise recall bias and focus on exploring adult cognitive processing of life events (age 10 is the threshold for criminal responsibility in England and Wales). We defined a close contact as “a relative or friend who mattered to you, and from whom you were able to obtain support, either emotional or practical”. This wide definition permitted inclusion of the ‘hidden bereaved’ (i.e., secret relationships, people at the fringes of the deceased’s social networks, people who hid their grief, etc.), and all those in the deceased’s social networks. We operationalised sudden bereavement as “a death that could not have been predicted at that time and which occurred suddenly or within a matter of days”. We ascertained exposure status using responses to the question: “Since you were aged 10 have you experienced a sudden bereavement of someone close to you due to any of the following: (a) sudden natural death (e.g., cardiac arrest, epileptic seizure, stroke); (b) sudden unnatural death (e.g., road crash, murder or manslaughter, work accident); (c) suicide?” We relied on the respondents’ subjective definition of cause, rather than the coroner’s verdict or death certificate, as our exposure of interest was perception of bereavement type.

### 2.3. Materials

An online questionnaire [10] was designed by Alexandra Pitman, Fiona Stevenson, David Osborn, and Michael King We consulted a group of young bereaved adults and bereavement counsellors, who suggested which domains to cover and advised on wording, and we piloted the questionnaire nationally [10]. 

Following 119 fixed-response questions eliciting quantitative data on socio-demographic and clinical characteristics, 20 open questions probed specific dimensions of the impact of bereavement. Of these, two explored the experiences of help received after the death:

“*What are your views on any help you were offered or not offered? In your response you may wish to comment on:*

- how helpful or unhelpful any support was
- what help you wish you had been offered and at what stage
- why certain people did not offer their support”

“*After the death did it feel as though support was available to other people close to that person but not to yourself? For example this may have been because:*

- you hid your grief
- others were not aware that you had a close relationship with this person
- the support you wanted was not available”

There was no upper word limit on free-text responses. The respondents were invited to give as much or as little detail as they wished or to skip the question. 

### 2.4. Ethics

Information provided to the participants explained that the study was being conducted by a UCL research team, including psychiatrists (Alexandra Pitman, David Osborn, and Michael King) and a medical sociologist (Fiona Stevenson), and that data would be anonymised and handled in accordance with data protection legislation. All the participants provided online informed consent. The study protocol was approved by the UCL Research Ethics Committee in 2010 (reference: 1975/002). 

### 2.5. Data Analysis

We imported responses to the two questions into Microsoft Excel, which allowed us to organize, review, and code large volumes of paired qualitative data. 

We used thematic analysis [28] to analyse free-text responses to the two questions on experiences of support, restricting our analysis to the respondents bereaved by suicide. Analysis was conducted collaboratively (by Tanisha De Souza, Adelia Khrisna Putri, Alexandra Pitman, and Nicola Morant) with the support of Microsoft Excel. After independent data familiarisation and discussions about emergent codes, the first 100 responses were coded independently by two members of the team (Tanisha De Souza and Adelia Khrisna Putri) and compared for consistency of coding. Discrepancies were resolved through team discussions, and further amendments to the coding framework were made. Tanisha De Souza then coded the full dataset, with regular team discussions of successive coding frameworks to clarify the organization and conceptual meanings of themes within higher-order categories. We reviewed the data against our final coding framework to validate our analysis. We reported our findings following COREQ guidelines [29].

To present descriptive characteristics, we used Stata version 12 (StataCorp., College Station, TX, USA), comparing characteristics for the qualitative sample with those for the wider quantitative sample of suicide-bereaved adults.

The dataset generated and analysed in the current study is not publicly available due to the risk of identifying the participants. Requests to analyse these data should be made to the corresponding author, subject to honorary contract status approval in the UCL Division of Psychiatry. 

## 3. Results

### 3.1. Sample Characteristics 

Of the estimated 659,572 bereaved and non-bereaved people receiving the email invitation, 5085 people responded to the questionnaire by clicking on the survey link, and 4630 (91%) consented to participate in the online study. An overall response rate could not be accurately measured, as the precise denominator of people bereaved by suicide in the UK is not obtainable using routine data or survey methods. Of the 3432 participants meeting the inclusion criteria, a total of 614 people had been bereaved by suicide. We analysed data for the 420 (68%) suicide-bereaved individuals who provided responses to one (*n* = 121) or both (*n* = 299) of the two questions on experiences of support. 

This sample of 420 respondents was predominantly female (83%) and of white ethnicity (92%) with a mean age of 25.4 (standard deviation = 6.1), and the sample was balanced between those bereaved by a relative (49%) *versus* non-relative (51%). Over half (61%) had attained degree-level qualifications, and 62% were classified as social classes 1 or 2. These characteristics did not differ substantially from those of our larger sample of total survey respondents (see Table 1). 

In their quantitative responses the majority of respondents (75%) reported having received any kind of bereavement support, most commonly informally from friends or family (62%). Most (86%) had found the support valuable in coping with grief, although many of the comments indicated a desire for more support. Almost half (41%) of the respondents who reported support had received it ‘formally’ from a professional, most commonly a healthcare practitioner, therapist, or counsellor in the statutory, private, or voluntary sector (see Table 2). 

### 3.2. Qualitative Findings

Through the process of thematic analysis, we organized findings into three broad descriptive areas corresponding to aspects of support judged by the respondents to be important after suicide bereavement (see Table 3): value and experiences of the support received; views on specific support needs; and reasons for not seeking support. We present details of each of these below, with illustrative examples. These are given as typed in the online responses, except where spelling errors have been corrected. Some responses were coded in more than one thematic area, and we have indicated where themes overlapped. Percentages are given as a guide to the prevalence of the receipt of different support, as indicated in quantitative responses (see Table 1 and Table 2).

#### 3.2.1. Value and Experiences of the Support Received

In this section, we provide a descriptive overview of the respondents’ experiences of four forms of support: informal support in existing social networks; support from others with similar bereavement experiences; professional support; and practical support (spanning the previous three). These capture the tension between friends acknowledging the loss *versus* ignoring it, the polarised views about the value of professional support, and unmet needs. 

##### Informal Support Experiences

Responses indicated that family and friends were the most valued sources of support, and this was provided throughout the extended social network, including neighbours, colleagues, religious communities, and the relatives of the deceased. Specific factors appreciated were: receiving emotional support whenever needed, knowing that people were there if needed (latent support), not being treated differently, and having opportunities to talk about the deceased openly. The concept of being treated ‘normally’ arose frequently and was valued because it countered the stigma of suicide. However, this meant different things to different people, reflecting individual personality and interaction styles or support needs rather than gender differences. To some, it meant friends acknowledging their loss and offering them the opportunity to talk, thereby demonstrating acceptance. To others, it was preferable for people not to talk about the loss at all to avoid treating them ‘differently’ and to avoid intrusiveness. Where these two positions met was in the general agreement that others’ social awkwardness on the topic was extremely uncomfortable and frustrating, adding to the burden of the loss. The communication difficulties this created were perceived as a barrier to receiving beneficial informal support.

“*The support of my close friends was helpful because I was able to talk openly about what happened and discuss distressing thoughts … I also appreciated the concern shown by… some of my work colleagues. Other work colleagues did not mention what had happened at all to me. I knew that everyone had been told what had happened and the fact that they weren’t talking to me about it made me think that they were probably talking about it behind my back. I think that people might have been scared of saying the wrong thing or upsetting me*.”

(33-year-old woman, bereaved seven years previously by the suicide of her grandfather)

“*I am very annoyed at people for not offering concern or support at all after her death but I understand that they might feel awkward or afraid to bring up this issue*.”

(20-year-old woman, bereaved two years previously by the suicide of a close friend)

“*I think I just needed people to not me treat me differently or stigmatise me because of the death. It varies from person to person what kind of help people need, but I felt as though I was mainly able to get over it on my own and didn’t need to talk about my feelings all the time*.”

(20-year-old woman, bereaved five years previously by the suicide of her father)

“*the support offered was not suitable, because I didn’t wish to discuss it and that was all they were interested in.*”

(30-year-old man, bereaved 11 years previously by the suicide of his partner)

“*I didn’t want to talk about it. It’s nice to know people are there for you. Everyone says the same things like ‘It’s not your fault’ etc. which doesn’t really help at the time but I guess you need to hear it even though it seems cliché*.”

(20-year-old man, bereaved a month previously by the suicide of a close friend)

Although gender differences were not apparent in preferences for being communicated with, they were recognized (by both male and female respondents) in the manner that peers responded to their loss. Generally, men were perceived as less comfortable addressing the topic.

“*I lived with 5 other males when I found out. I don’t think guys are very keen to elicit signs of weakness in their friends (i.e., ask them things that might make them emotional). This meant they did not really ask how I was. Support in this sense was therefore non-existent. … One of my female friends asked how I was doing quite a lot after I told them. This was helpful*.”

(25-year-old man, bereaved three years previously by the suicide of a close friend)

“*I noticed men usually avoid saying anything to you whereas girls are better at saying something. I told my housemate at the time it’s better to say something, anything rather than nothing*.”

(35-year-old woman, bereaved seven years previously by the suicide of her father)

The polarity of preferences regarding others’ responses highlighted the need to gauge a bereaved person’s preferences for emotional support after a suicide, balancing intrusiveness with finding a way to acknowledge the loss appropriately. Taking these responses together, general advice on how to support someone bereaved by suicide included open communication, accessibility, and latent support.

“*The most helpful support I received was from my friends who simply carried on as usual while letting me know I could talk about whatever I wanted, whenever I wanted*.”

(24-year-old woman, bereaved nine years previously by the suicide of her brother)

Beyond these communication difficulties, many participants felt let down or resentful towards those in their networks who were unwilling or unable to provide help. Some who did receive informal support reported feeling judged, a burden, or too guilty to ask for more. This was one of the reasons that some had not sought further support, discussed in more detail in Section 3 below. 

##### Shared Experiences of Suicide Bereavement

The opportunity to discuss and share feelings with others had also been bereaved by suicide was highly valued by many participants, whether with relatives and friends bereaved by the same suicide or strangers contacted through the voluntary sector. This perhaps reflected a lack of social awkwardness among those with similar experiences and the sense of not feeling different.

“*help and support was offered between the members of the family and it felt like a period of bereavement and grief that we went through all together*.”

(32-year-old woman, bereaved 17 years previously by the suicide of her aunt)

“*to have some common friends with the person that died helped… because it made me feel less lonely in my pain*.”

(36-year-old woman, bereaved seven years previously by the suicide of a close friend)

Of those who sought support through voluntary organisations (11%), this was primarily from bereavement support groups. All participants who reported utilising this type of support described positive experiences, valuing the opportunity to befriend and receive emotional support from people who had also experienced suicide bereavement.

“… *through the internet I found some support services such as Survivors of Bereavement by Suicide (SOBS). The support offered by SOBS was brilliant and it was nice to speak to people who had been through similar experiences. I also joined some online website support services which I have made friends with two young women (in their 20s) whose fathers also committed suicide and I found being able to talk to them very comforting and beneficial*.”

(27-year-old woman, bereaved seven years previously by the suicide of her father)

##### Professional Support Experiences

Even with good support from family, friends, or bereaved peers, many individuals also expressed a need for professional support or the comfort of knowing that this support was available. This was also seen as a way of lifting the burden on family and friends. Knowing that professionals were there if needed mirrored the value placed on latent support from family and friends. In both contexts, being offered support, even if it was not taken up, seemed to communicate an acceptance of the bereaved person’s difficulties. With that initial offer established, communication could then continue without awkwardness. 

However, only a minority of our sample reported actually accessing formal support, whether through healthcare services (16%) or from private counsellors or therapists (13%). Many others expressed a wish that some form or counselling had been offered. Low levels of GP consultation may have reflected a fear of being prescribed medication or of wasting professionals’ time.

“*I didn’t want to go to my GP as I felt I would waste their time, I was embarrassed and was scared they would prescribe me antidepressants or sleeping tablets because I didn’t want to rely on tablets to get me through my day. My first week at uni however I felt like I couldn’t cope anymore and went to the university health centre. When telling the GP what was wrong it really helped me. it was like breaking down in front of him released some of my emotions and knowing I could come back if I felt worse reassured me that I did have support available*.”

(20-year-old woman, bereaved two years previously by the suicide of her father)

“*Only offered anti-depressants & sleeping pills but did not want to avail of these as had a young son to care for*.”

(34-year-old woman, bereaved 10 years previously by the suicide of her partner)

Some people reported excellent care from their GP, specifically valuing acknowledgement of grief and being signposted to local bereavement support. However, others felt that their GP had lacked empathy, failed to pick up on cues (also covered below under Need for Proactive Support), or had overlooked the significance of the bereavement in relation to physical or psychological complaints.

“*The G.P could have picked up on my anxiety better as the inhaler did nothing to help. Wish he would have helped more, offered me other support etc*.”

(26-year-old woman, bereaved four years previously by the suicide of her uncle)

“*I was rather appalled at my GP at the time … At some point, I said ‘well, I have been through a lot lately emotionally’ and hoped she would pick up on that, I was thinking at that point that maybe bereavement counselling wouldn’t be bad, but couldn’t get myself to ask for it. She didn’t respond, the moment passed and that was it*.”

(38-year-old woman, bereaved 10 years previously by the suicide of her mother)

The respondents expressed very mixed experiences of counselling, which seemed to vary by whether they had established rapport with a specific counsellor. Where rapport had been gained, generally the positive aspects of counselling included being able to discuss any aspect of the bereavement, having a space to express grief, taking the burden off friends and family, and feeling comforted by a compassionate yet objective approach. In some cases, having emotions normalised or validated by an experienced professional was specifically valued.

“*I had counselling a few years later and it was really good to talk about it. It was only then I could make sense of how I was feeling. It was useful to hear that it was normal to feel like this*.”

(32-year-old woman, bereaved 15 years previously by the suicide of a close friend)

“*I was at university at the time and their counselling service were fabulous and offered help straight away - it was very cathartic to be able to go into a room with a stranger and cry when I felt as is my close confidants needed a break from it. It was all very raw then and I did not know until later on how very isolating and unusual suicide is and how his death would affect certain areas of my life*.”

(36-year-old woman, bereaved 15 years previously by the suicide of her partner)

Negative experiences with counsellors related to perceptions of the counsellor as patronising, judgmental, or unable to connect due to generational differences.

“*The university asked me to go see a counsellor. I went to one session, but didn’t really find it very useful. The lady had nothing in common with me or my family (being aged about 70) and it was almost as if she disapproved of what my brother did*.”

(25-year-old woman, bereaved six years previously by the suicide of her brother)

“*I often felt that counsellors were patronising and disliked them a lot. I felt they offered no support at all and were more interested in mine and the deceased relationship than helping me overcome the past and look to the future*.”

(20-year-old woman, bereaved four years previously by the suicide of a close friend)

“*I was pleased to be offered a counselling session as would not have thought of this myself. I didn’t like the counsellor and thought the things he said were trite but maybe that was inevitable under the circumstances. I decided not to attend any more sessions*.”

(36-year-old woman, bereaved 15 years previously by the suicide of a close friend)

Negative experiences of counselling countered the suggestion (illustrated below) that counselling should be mandatory, by highlighting the problem of acceptability. This was perhaps particularly relevant for men given the gendered disdain for counsellors expressed in Section 3 under Lack of Faith in Available Support. Feeling pressurized to see a health professional was also described as very uncomfortable, and contributed to negative experiences of professional support.

“*We had the choice to see the school counsellor but I didn’t go because I was too shy. Eight years later, after her only brother committed suicide, I decided to see the university counselor. I wish they made it mandatory to see the counselor because I had no support at all from home, but was too shy to ask for it*.”

(25-year-old woman, bereaved 11 years previously by the suicide of a close friend)

“*My local doctor was very unhelpful, I felt pressured to go and see her and I felt like she was intruding on my grief*.”

(22-year-old woman, bereaved 12 years previously by the suicide of her father)

Views over the optimum timing of professional support varied greatly, again as per individual circumstances, and this is covered in more depth in Section 2. Generally, unhelpful encounters with healthcare professionals, managers, or teaching staff created or reinforced perceptions that people did not care or understand what the bereaved person was going through. These negative experiences of trying to access support had a marked impact in disincentivising future help-seeking behavior, therefore linking to Section 3 on reasons for not seeking support. Unmet emotional needs for formal support from counsellors or health professionals included training for health professionals in responding to suicide loss, and improving awareness of relevant local bereavement support.

##### Practical Support

Several people valued having had their practical needs acknowledged after the bereavement, whether by professionals, relatives, or friends. This included help with childcare or paperwork, absences from work or school, postponed deadlines, signposting to legal or financial advice, and practical advice on managing emotions. Assistance from employers or teaching staff in agreeing time off, deadline extensions, or mitigating circumstances for exams seemed to be particularly valued, especially when navigating what was seen as a “*ruthless*” system of educational regulations.

“*School were brilliant—didn’t hassle me for not going to lessons, extended all my deadlines, let me drop some lessons, but had meeting with tutor once a week to see how I was getting on, but (nicely) encouraged me to start doing work when the exams were coming up … really helpful*.”

(21-year-old woman, bereaved four years previously by the suicide of her sister)

However, some respondents described a notable lack of practical support from informal networks and professionals. They felt disappointed that people had not recognised and responded to what they had felt were very obvious unmet needs. This was despite the apparent advantages of practical help bypassing having to talk about emotions.

“*The best help that I could have had would have been help from family and friends to do my housework and look after my children. I felt so tired because my baby did not sleep at night and I was pregnant and breastfeeding*.”

(30-year-old woman, bereaved six years previously by the suicide of her brother-in-law)

“*The paperwork I had to go through to show why I hadn’t completed some uni work made me feel extremely guilty and like I was using it as an excuse. I would have rather spoken about it more with someone who would sort it out for me … at a time like that, the last thing I wanted to do was fill in a blanket document that covered all forms of ‘mitigating’ circumstances’ at uni*.”

(21-year-old woman, bereaved one year previously by the suicide of a close friend)

#### 3.2.2. Views on Specific Support Needs

The respondents suggested specific ways in which they felt their emotional and practical support needs could have been better addressed. These included the examples given above in Section 1, the need for support to be offered proactively and repeatedly over time, and a warning against making assumptions about how affected someone might be after a suicide. 

##### “Legitimacy” of Support Needs 

Several participants described how perceptions of ‘legitimacy’ could hinder access to or receipt of support, especially when the bereaved person was regarded as ‘less close’ to the person who died than others in the social network. This feeling that they were not entitled to support, whether formal or informal, arose either because they viewed themselves as less worthy of support than others, or because other people viewed them this way. Where others were perceived to have made this value judgment, it was assumed that the bereaved person was not sufficiently close to the person who had died or was not sufficiently affected by the death. Many respondents described others’ perceptions of a hierarchy of need governing access to support, with close family generally at the top and friends and colleagues further down. Some respondents reported that young children were also judged as low in this hierarchy, based on beliefs about being too young to grieve. Such value judgements were regarded as unjust, relying solely on others’ perceptions of a bereaved person’s relationship to the deceased or the extent of their grief. A key resentment was that people sometimes made assumptions about closeness based on their presumptions about the link between relationship labels and degree of intimacy. 

These comments were most commonly expressed by non-relatives, who often cited this as a reason for being offered no support by healthcare professionals, employers, or teachers. Such groups, as well as the deceased’s relatives, were seen as those who most frequently made these judgements. Comments suggested that these people often felt they had the authority to decide who was worthy of support and at what stage. Close friends felt angered and upset when their relationship with the deceased was not acknowledged, thus missing out on support available to the deceased’s relatives. Others in the more extended social network, particularly ex-partners, colleagues, compartmentalized friends, and ‘hidden’ relationships also felt left out, resentful, or undeserving of help.

“*No support was offered to me, however it may have been to other friends at his school or to his family. I don’t think I was deemed to be ‘close’ enough to him*.”

(25-year-old woman, bereaved 10 years previously by the suicide of a close friend)

“*I tried talking to his family, but it was as if I wasn’t allowed to grieve for him because they thought I wasn’t as close to him as them*.”

(19-year-old woman, bereaved two weeks previously by the suicide of a close friend)

Although family members were generally viewed by others as being prioritised for support, such hierarchies were also applied within families. These tended to deem partners or parents as in the greatest need, leaving extended family members relatively neglected.

“*I felt it was all aimed to the brother and sister and parents of my cousin and no-one really cared how I felt*.”

(20-year-old man, bereaved five months previously by the suicide of his cousin)

“*I felt like everyone was rallying round after my stepdad who was playing the role of the grieving widow(er). Having meals cooked for him etc. And family friends in the village sort of forgot about my brother and I*.”

(27-year-old woman, bereaved four years previously by the suicide of her mother)

Age was also regarded as a factor used by others in judging needs for support. In many cases respondents bereaved in childhood felt that they had been viewed as too young to have been affected by the suicide or to have understood the circumstances surrounding it. They felt this had greatly limited their access to both formal and informal support.

“*As a child/young person, people don’t take your emotions seriously. There should be more help available so that the child learns how to deal with emotions, so that they don’t come later on in life*.”

(28-year-old woman, bereaved 17 years previously by the suicide of a grandparent)

In some cases, such value judgements had been made by the respondent themselves, who deemed him- or herself less worthy of support than others. By putting others’ needs first and hiding their grief, these individuals often missed out on support.

“*I felt that my mum got the most support, which was rightly so. I hid a lot of the grief and so people thought I was fine and didn’t need support*.”

(28-year-old woman, bereaved four years previously by the suicide of her sister)

“*I felt I didn’t have the right to grieve her and that that was why there was not support for me*.”

(26-year-old woman, bereaved four years previously by the suicide of her aunt)

##### Need for Proactive Support

Regardless of whether the respondents felt they been well-supported, a common theme was the need for friends and professionals to be more proactive in offering help. This seemed more applicable to formal support, because people reported feeling less comfortable asking for help from professionals than from social networks. Moreover, there was a widely-held expectation that relatives, friends, employers, teachers, and healthcare professionals (particularly GPs) had a responsibility to help the bereaved person access appropriate resources. This applied particularly in the immediate aftermath of the loss, at a point where people reported feeling too distressed to seek help on their own or to act on any information provided. Many felt let down by GPs, friends, or relatives who did not intervene at this difficult early stage to help them access support or to communicate latent support. GPs’ failure to pick up on cues was also mentioned above in Section 1, and this was seen as a crucial missed opportunity to offer support,

“*I feel that if my GP had perhaps directed me immediately to a bereavement counsellor this would have been better. I certainly would have benefited from somebody giving me direct instructions as to how to find this support, as I was so emotionally exhausted that I could not find the help I needed myself*.”

(24-year-old woman, bereaved one year previously by the suicide of her brother)

Some respondents who had managed to access counselling expressed regret that they had not done this earlier or even resentment at having had to do this on their own initiative. They felt that a referral or encouragement by their GP, friends, family, employer, or teaching establishment could have offered them the benefits of counselling at a much earlier stage. Even if not ready to accept help at that point, it was felt that outlining what was available would have planted the idea that the option was there for when they were ready.

“*I sought professional help myself—but that was only very recently. I wish there had been some sort of counselling or service that offered their assistance… Although I knew there were services available it was still too raw for me to actively seek anything for myself*.”

(27-year-old woman, bereaved five years previously by the suicide of a cousin)

“*once I finally found the appropriate help for me individually I found it very helpful and would recommend it to anybody … I wish that the (counselling) was offered to me sooner as I think that it would of helped me more by encouraging me to go back to lessons and I think maybe I wouldn’t have missed out on so much or dealt with the situation in ways that I did*.”

(19-year-old woman, bereaved almost two years previously by the suicide of a close friend)

“*I wish I could have had counselling or something sooner—it took counselling ten years after his death to realise that I needn’t feel guilty and I couldn’t have influenced it*.”

(29-year-old woman, bereaved 15 years previously by the suicide of her uncle)

##### Early and Repeated offers of Support over Time 

Overall, the respondents’ mixed views about the appropriate timing of formal support suggested that it should be offered at an early stage, and then, repeatedly, so that help could be taken up when the individual felt ready. Although early and proactive offers of support were felt by many to be important, whether providing actual or latent support, some people felt that they had been too distressed at this early stage to accept professional help as the loss had not yet “*sunk in*”. Although a number of these individuals went on to access support later on, some considerably later, many found it difficult to access help at that stage, because others expected them to have recovered.

“*I was offered help straight away from the police in regard to counselling. You don’t need it straight away, you need it much later and no one offers it to you then. You also feel reluctant to ask for help yourself later, because it has been so long and you feel that you should have dealt with it by then.*”

(28-year-old woman, bereaved four years previously, by the suicide of her sister)

“*Support was offered but it was too soon at the time. Wish that 3 months later people would still have recognised that help was needed and offered it to me. Up until that point I was too scared to talk about it, as it made it real. I know that I could have sought out help but was too scared.*”

(20-year-old woman, bereaved four years previously by the suicide of her father)

#### 3.2.3. Reasons for not Seeking Support

A quarter of our sample had received no support after the suicide, but their reasons ranged from a lack of need or low expectations of deriving benefit, to a yearning for support yet difficulty or a fear of asking for it. Negative experiences of trying to access support, as described in Section 1, disincentivised reattempts. This was particularly true where peers and relatives failed to acknowledge the loss, or professionals missed key cues. What was not clear was whether such individuals chose to ignore support needs or were simply unaware of them.

##### Reluctance to Express Grief

A large minority of the respondents reported hiding their grief, most commonly due to stigma and a fear of being judged, perceptions of low entitlement, putting others’ needs first, or conflicting pressures of work, education, or childcare. Despite cultural assumptions about gender in relation to talking about emotions, our finding of a reluctance to express grief was not apparently gendered. 

Generally, hiding grief appeared to have hampered access to support from professionals, friends, and family. Fears of being judged or stigmatised related to the anticipation that people might treat them differently or feel awkward if they expressed their true emotions. A fear of being pitied was mentioned, as were cultural expectations about concealing emotions.

“*I tended to hide my grief because I didn’t want to stand out because of it at school. I didn’t want anyone to know because I didn’t like that kind of attention. I didn’t want anyone to have sympathy on me so I tended not to share this with anyone, including those whom I considered my good friends*.”

(24-year-old woman, bereaved 11 years previously by the suicide of her father)

“*I hid my grief and cried in private because in the end you just get labelled anyway. There’s a real problem in this country showing any kind of emotion is seen as wrong or soft*.”

(38-year-old woman, bereaved six months previously by the suicide of a cousin)

“*it is hard to look for help because of the stigma attached to it.*”

(20-year-old woman, bereaved one year previously by the suicide of her brother)

In some cases, hiding grief was linked to a belief that others were more deserving or that others might judge them not to be entitled to grieve after the death, as we have described in Section 2 under Legitimacy.

“*Other people had much closer relationships and were more deserving of help*.”

(32-year-old woman, bereaved 10 years previously by the suicide of an uncle)

“*I hid my grief because I didn’t think I had as much of a right to mourn him because I was the one who had taken the decision not to be in a relationship any more. I still loved him as a friend though which is why it hurt so much*.”

(25-year-old woman, bereaved two years previously by the suicide of an ex-partner)

Several people described responsibilities to others as the reason for suppressing their own grief. In hiding their grief, they wanted to appear strong for the sake of others or felt expected to provide support, therefore sacrificing their own needs. Younger generations often felt they ought to support their parents emotionally or described the expectations of parents that their children (primarily men) should support them by taking responsibility for running the household. In some cases, practical conflicts such as work, parenthood, or educational commitments were barriers both to grieving openly and to accepting or seeking support.

“*I stopped (counselling) around the time of my exams as it was better to ignore what had happened and concentrate on revising, than to have everything stirred up*.”

(27-year-old woman, bereaved four years previously by the suicide of her mother)

“*I don’t ever remember being offered support. My mother seemed to think it was my place to support her (this applies to all the deaths we have experienced)*.”

(26-year-old man, bereaved six years previously by the suicide of a family friend)

As with others who described suppressing their needs and seeking support long after the death, it was often harder to access support at a later point. Many reported having to hide their grief out of fear of being judged for not having ‘moved on’, or because they berated themselves for not having ‘recovered’.

“*I hid my grief and because my mum was having a nervous breakdown and suffering from depression I felt like it was impossible for me to be vulnerable also. I had to look after my younger sibling and manage the house. I’m sure there was support available but when I was ready for the help nobody offered it*.”

(20-year-old woman, bereaved four years previously by the suicide of her father)

##### Low Perceived Need

Only a small minority of the respondents commented that they had not been affected by the death or did not want any support, formal or informal. The method of data collection meant that their reasons for this were not often clear, nor were their alternative coping mechanisms. However, choosing not to access support did not always mean someone was not emotionally affected by the death. Sometimes avoidance of help was due to a fear of being pitied, a walling off of grief, a fear of showing weakness, or perceived responsibilities to others. Again, intergenerational expectations of young adults supporting their parents caused those individuals (generally male) to subsume their own needs for the sake of family members.

“*I have always been a closed and individual person so I did not seek help at any time. The last thing I want is my friends thinking ‘That’s awful, I feel for her but I wish I knew how to politely change the subject”‘…. Everyone has problems, I’m no different*.”

(20-year-old woman, bereaved four years previously by the suicide of her father)

“*Since I kept myself to myself and mostly shunned the death as a whole, I felt I did not require any help… I hid my grief, and so did the rest of my family, it was as though the ordeal was embarrassing to the family as a whole. But since I considered him selfish for his actions, I did not want to grieve him*.”

(18-year-old woman, bereaved 11 months previously by the suicide of her uncle)

“*My job at the time was to look after my family and ensure they had the opportunity to deal with their grief. There are limited finances and it was more important to me that they had access to the help they needed*.”

(30-year-old man, bereaved 11 years previously by the suicide of an unspecified family member)

##### Lack of Faith in Available Support

Overlapping with perceptions of low perceived need, a minority of respondents expressed skepticism about the effectiveness of the support offered and felt that they alone could deal with their grief. Some conveyed a sense of hopelessness about their situation and the inability of anyone, professional or otherwise, to alleviate their inevitable suffering. This was sometimes reinforced by negative experiences of unhelpful support. The nihilism we observed was not exclusive to men. However, a specific disdain for professionals was more apparent in men, who appeared to fear the stigma of requiring psychological help or who explained that counselling was not “*how I deal with things*”.

“*Help suggests that something can be made better. When your brother kills himself, it cannot be made better. Could someone help me to feel better? I don’t want to feel better. My brother is dead*.”

(32-year-old man, bereaved 10 years previously by the suicide of her brother)

“*I tried to have counselling straight after but there wasn’t anything that anyone could do about my situation, I just had to get on with it*”

(27-year-old woman, bereaved four years previously by the death of her mother)

“*I didn’t want help, and still don’t, and am glad I didn’t get offered any by ‘professionals’*.”

(20-year-old man, bereaved three years previously by the suicide of a close friend)

“*Help was offered by doctor immediately, but I declined as I saw counselling as a sign of weakness on my part*.”

(29-year-old man, bereaved 14 years previously by the suicide of his father)

Where respondents had learned to avoid asking for support because they felt let down by their experiences of seeking help, this underlined the importance of offers of support being repeated and in providing a range of options.

## 4. Discussion

### 4.1. Main Findings

Our findings indicate that informal networks are the main source of support for people bereaved by suicide and that, where lacking, this can reinforce feelings of abandonment. Having access to a broad range of support, including peer support, is greatly valued. Knowing that latent (or potential) support existed is comforting, even if offers are not taken up. GPs and other health professionals are seen as having an important role in being proactive to help bereaved people access appropriate specialist support as early as indicated. The most positive aspects of professional support identified were being able to talk openly about the death, being directed to appropriate resources, and discussing feelings with an objective individual.

However, some respondents, even those who had access to formal support, expressed resentment about feeling let down by close friends and family, who were not perceived as providing emotional or practical help. This often related to others’ difficulties in broaching the topic, knowing what to say, or treating them differently. Negative experiences of formal support commonly related to professionals lacking empathy, missing cries for help, or avoiding talking about the death. Perceptions of stigma or a lack of entitlement to help seemed to be a major barrier to help-seeking, particularly where reinforced by others. Friends whose relationship with the deceased went unacknowledged felt shortchanged, with support only available to the inner circle of contacts— primarily relatives. Social pressure to hide grief also concealed needs for support and was often linked to stigma. 

Themes varied little by age except where adult children carried an expectation of putting parents’ needs before their own. Themes varied only by gender in relation to a male disdain for professional help and in observations that male peers found communication more awkward. Gender differences were apparent in parents’ expectations of sons to take over the household, more than sons’ expectations to do so. However, kinship differences were apparent in friends’ and distant relatives’ unmet needs for emotional support and their sense of having the legitimacy of their support needs questioned. The relatively young age of our sample may explain the prominence of this theme for non-relatives. Transition to young adulthood involves the establishment of a friendship networks that can often feel stronger than family ties, particularly in contexts of intergenerational disenfranchisement. Where bonds with the deceased were very close, peers would be expected to experience intense grief. Not having those bonds acknowledged could add to that grief, compounding loneliness and presenting a barrier to support.

### 4.2. Findings in the Context of Other Studies

The findings of this study complement the quantitative findings from this same dataset of a lower likelihood of receiving informal support after suicide and delays in receiving support compared with people bereaved by other sudden deaths [20]. The experiences of social awkwardness described are also consistent with the findings of our qualitative interview study (recruited from the same wider sample) exploring the experience of stigma after suicide loss. In this study, a key theme was social awkwardness around the topic of suicide, to the point of others’ social avoidance and failure to offer support [19]. The social ineptitude of others after suicide was also described in a Norwegian qualitative study [30]. Qualitative findings from Britain [31], Australia [22,32] and African-American communities [33] further identify stigma as a significant barrier to accessing appropriate support after suicide bereavement. What seems to set suicide bereavement apart from other losses, is that whilst any bereavement is an individualized, contextualized, and multifaceted experience [34] with support needs proportionate to the severity of grief [35], suicide carries a particular burden in others’ discomfort and avoidance [19]. 

As in the current study, a number of smaller-scale qualitative studies have emphasised the importance of social and peer support in the aftermath of a suicide [21], including Irish [36,37], North American [38,39], and Australian qualitative work [22]. Our findings regarding the need for others to initiate support are similar to those from Australian [22,40] and Danish qualitative work [41] and our previously published British focus group discussion [42]. Irish research participants bereaved by suicide specifically identified a central role for their GP [23]. The respondents in our study sometimes described a lack of empathy in friends or professionals, but not the tactlessness or insensitivity reported in one Australian study [22] or the aversion to peer support groups described in another Australian study [40]. Together this evidence suggests that poor mental health outcomes after suicide bereavement may be related to experiences of lack of support, whether due to self-stigma [20] or others’ ineptness in judging and meeting their needs [19].

### 4.3. Strengths and Limitations 

Our study will inform the implementation of a key objective of the suicide prevention strategy: better support for people affected or bereaved by suicide. To our knowledge, it is the largest British study using a defined sampling frame to explore young adults’ views and experiences of help received after suicide bereavement. Using direct email invitations and a closed online survey, we could be clear about our population of interest and therefore the limits of generalisability. We analysed data collaboratively and regularly discussed our emerging coding framework to enhance validity and consistency of coding. As per recommendations on team approaches [43], our team’s mix of researchers from psychology, psychiatry, and sociology perspectives helped us explore differences in interpretation and reduced the influence of theoretical or personal preconceptions.

Whilst avoiding the biases inherent to sampling a help-seeking population or using an open survey, the method of recruiting from HEIs may have introduced selection bias by favouring those in higher socioeconomic groups. Our sample was primarily young, female, and white, and predominantly students, and these factors limit the generalisability of our findings. Response bias from those who had received the least support is also possible, as is recall bias of distant bereavements. The wording of our two questions on this topic may have been viewed as leading, in assuming negative experiences. However, this issue was specifically suggested by our consultation group as an important dimension to explore. Without interviewer presence it was otherwise difficult to probe support experiences using a more neutral question. Although our sample was large, the low volume of online data from each participant meant we sometimes lacked context for statements and had no opportunity to probe meaning. Some participants only responded to one of the two questions on support or gave vague or ambiguous answers. This may have been due to response fatigue or a lack of strong views. A questionnaire solely focusing on the experiences of support received after bereavement by suicide may have been more effective in soliciting in-depth, more meaningful answers from the participants. 

### 4.4. Clinical and Policy Implications

The provision of support to people bereaved by suicide continues to be a priority within prevention strategies [44]. Young and middle-aged men are the demographic group at greatest risk of suicide [44], and support after exposure to peer suicide may be particularly important for them. Our findings from bereaved relatives and non-relatives confirm that needs for emotional support are distributed throughout the social networks of people who die by suicide, and assumptions should not be made about entitlement based on perceived closeness. Failure to acknowledge support needs after a relative or peer’s suicide may contribute to social exclusion, deteriorating mental health, and learned helplessness. This does nothing to mitigate the potential for hopelessness and suicide suggestion [45]—both influences on suicide risk. Suggested policy responses include systems by which health professionals or institutions actively seek out those affected by a suicide to offer support to counter any avoidance within their social networks. National campaigns to advertise the range of support sources available would also promote help-seeking in anyone perceiving need. 

In England, responsibility for implementing the national suicide prevention strategy [46] lies with local public health agencies, and local suicide prevention plans are expected to include provision of support for people bereaved by suicide [47]. The conundrum is that few trials have been conducted on specific interventions for this group [48], and the evidence base is weak [5,48]. The current study is one of few that identify which interventions are likely to be most acceptable, feeding into the design of trials. Candidate interventions, as suggested by this study, include training healthcare professionals, teachers, university staff, and employers to be proactive in meeting practical and emotional needs; improved internet resource hubs for those who feel unable to seek help; advice for those supporting someone bereaved by suicide on how to provide emotional and practical support; and improved signposting for GPs and the general public on how to access specialist bereavement support. Whilst in our study this was perceived as a key role for GPs, GPs themselves lack confidence in responding to the suicide bereaved [24] and should be prioritised for training. Similar outreach training for police, coroners’ officers, undertakers, and ambulance staff is also important as these professionals are most likely to have immediate contact with bereaved families and friends after a suicide [40]. 

Further research is needed to explore the support needs of ethnic minorities, older adults, and those in lower socio-economic groups. More in-depth work will be required to explore the responses of men [49] and specific cultural groups to suicide loss [33]. Until then, the current study points towards the need to develop systems of proactive support, with flagging mechanisms so that offers are repeated. Such systems will also need to address the needs of children and adolescents, in keeping with efforts to prevent the onset of mental health problems in childhood and to build resilience.

## 5. Conclusions

People bereaved by suicide value a broad range of emotional and practical support from family, friends, bereaved peers, and professionals. However, they often feel let down by the inability of others to recognise, understand, or respond to these needs, or even sometimes acknowledge the loss. The responses to our survey revealed hidden grief concealing extensive needs for support, which would have been uncovered if others had been more proactive in intervening. Better training for professionals and the lay public on how to respond to support needs is indicated, including advice on repeating offers of support should someone not feel ready early on. Such guidance should also discourage assumptions that non-relatives’ support needs are of lesser legitimacy. Addressing the unmet needs described in this study would help reduce distress and self-stigma and potentially reduce the observed risk of suicidality and mental health problems in this group. 

## Figures and Tables

**Table 1:** Demographic characteristics of the 420 respondents compared with the full sample of 614 people bereaved by suicide.

Characteristic	Full Sample of People Bereaved by Suicide *n* = 614	Qualitative Sub-Sample of People Bereaved by Suicide *n* = 420
**Gender**		
male *n* (%)	115 (19)	71 (17)
female *n* (%)	499 (81)	349 (83)
**Median age (IQR)**	23 (20–29)	23 (20–30)
**Work status**		
working in paid job *n* (%)	68 (11)	89 (21)
studying at college/university *n* (%)	526 (86)	197 (47)
both working and studying *n* (%)	20 (3)	132 (31)
neither (on leave/unemployed) *n* (%)	0 (0)	2 (<1)
**Educational attainment**		
educated to maximum A level *n* (%)	243 (40)	163 (39)
educated to degree level or above *n* (%)	359 (58)	257 (61)
**Ethnicity (binary categories)**		
white *n* (%)	562 (92)	388 (92)
non-white *n* (%)	52 (9)	32 (8)
**Any religious beliefs**		
Yes *n* (%)	429 (70)	291 (69
No *n* (%)	180 (29)	126 (30)
missing *n* (%)	5 (<1)	3 (1)
**Socio-economic status ^¥^**		
social classes 1 and 2 *n* (%)	380 (62)	255 (61)
social classes 3 to 7 & 9 *n* (%)	213 (35)	155 (37)
missing *n* (%)	21 (3.4)	10 (2)
**median age bereaved (IQR)**	19 (17–23)	19 (16–23)
**median time since bereavement, years (IQR)**	3 (1–7)	4 (1–8)
**Perceived social support**		
no lack of perceived social support *n* (%)	345 (56)	228 (54)
moderate lack of perceived social support *n* (%)	168 (27)	120 (29)
severe lack of perceived social support *n* (%)	100 (16)	72 (17)
**Marital status**		
married/civil union/co-habiting *n* (%)	200 (33)	145 (35)
single/divorced/separated/widowed *n* (%)	412 (67)	274 (65)
missing *n* (%)	2 (<1)	1 (<1)
**Closeness to the deceased**		
quite close *n* (%)	254 (41)	166 (40)
very close *n* (%)	359 (58)	254 (61)
**Gender of the deceased**		
male *n* (%)	433 (71)	299 (71)
Median age of the deceased (IQR)	26 (20–44)	27 (20–45)
**Kinship**		
father *n* (%)	86 (14)	64 (15)
mother *n* (%)	31 (5)	24 (6)
brother (including half-siblings) *n* (%)	46 (7)	29 (7)
sister (including half-siblings) *n* (%)	15 (2)	9 (2)
grandparent *n* (%)	11 (2)	9 (2)
uncle/aunt *n* (%)	49 (8)	30 (7)
niece/nephew *n* (%)	4 (<1)	3 (1)
cousin *n* (%)	54 (9)	39 (9)
close friend (including colleagues) *n* (%)	250 (41)	160 (38)
partner/spouse *n* (%)	23 (4)	19 (4)
ex-partner/ex-spouse *n* (%)	15 (2)	9 (2)
in-law/step/adoptive relation *n* (%)	23 (4)	19 (5)
missing *n* (%) *	7 (1)	6 (1)

^¥^ using the National Statistics Socio-economic Classification (NS-SEC) based on the Office for National Statistics Standard Occupational Classification 2010 (SOC2010); * all missing cases for this variable were identified as non-relatives apart from one who did not indicate kinship; IQR = interquartile range; SD = standard deviation.

**Table 2:** Specific bereavement support received.

Bereavement Support Received	Full Sample of People Bereaved by Suicide *n* = 614	Qualitative Sub-Sample of People Bereaved by Suicide *n* = 420
Any formal or informal/support received after suicide bereavement		
yes *n* (%)	441 (72)	315 (75)
no *n* (%)	148 (24)	105 (25)
missing *n* (%)	25 (4)	0
Formal/informal support perceived to be valuable (of those receiving support)		
yes *n* (%)	374/441 (61)	271/315 (86)
no *n* (%)	198/441 (32)	39/315 (12)
missing *n* (%)	42/441 (7)	5/315 (2)
Type of informal/formal support received (of those receiving support)		
formal only *n* (%)	68/441 (11)	53/315 (17)
informal only *n* (%)	220/441 (36)	143/315 (45)
both formal and informal *n* (%)	153/441 (25)	119/315 (38)
any formal support *n* (%)	221/441 (50)	172/315 (55)
any informal support *n* (%)	373/441 (85)	262/315 (83)
Specific formal support received		
Health services (e.g., doctor, therapist, nurse, counsellor) *n* (%)	83 (14)	67 (16)
social services *n* (%)	1(<1)	1 (<1)
private therapist *n* (%)	73 (12)	55 (13)
voluntary sector *n* (%)	51 (8)	44 (11)
police officers *n* (%)	45 (7)	37 (9)
funeral directors *n* (%)	51 (8)	38 (9)
coroners’ officers *n* (%)	35 (6)	28 (7)
school teachers or school counselling service *n* (%)	9 (2)	6 (1)
college tutors or college counselling service *n* (%)	19 (3)	15 (4)
line manager or employee counselling service *n* (%)	1 (<1)	1 (<1)
*Subtotal formal*	221 (36)	172 (41)
Specific informal support received *n* (%)		
friends and family	370 (60)	261 (62)
religious/spiritual advisor	10 (2)	4 (<1)
complementary and alternative practitioner	0 (0)	0
*Subtotal informal*	373 (61)	262 (62)
Other support received		
self-help (website, book, leaflet)	79 (13)	64 (15)
other (not classified as above)	1 (<1)	1 (<1)
Total	614 (100)	420 (100)

**Table 3:** Descriptive themes.

1. Value and experiences of the support received	Informal support experiences Shared experiences Professional support experiences Practical support experiences
2. Views on specific support needs	“Legitimacy” of support needs Need for proactive support Early and repeated offers of support over time
3. Reasons for not seeking support	Reluctance to express grief Low perceived need Lack of faith in available support
